# H_2_S donor S‐propargyl‐cysteine for skin wound healing improvement via smart transdermal delivery

**DOI:** 10.1002/mco2.485

**Published:** 2024-03-02

**Authors:** Xiaoqing Zhao, Yao Chen, Zhongxiao Lin, Xinyang Jin, Bolun Su, Xiaotong Liu, Mao Yang, Keyuan Chen, Menglin Zhu, Lei Wang, Yi Zhun Zhu

**Affiliations:** ^1^ State Key Laboratory of Quality Research in Chinese Medicine, Faculty of Chinese Medicine Macau University of Science and Technology Macau China; ^2^ Department of Medical Cosmetology Affiliated Hospital of Nantong University Nantong Jiangsu China; ^3^ School of Pharmacy Macau University of Science and Technology Macau China; ^4^ School of Medicine Macau University of Science and Technology Macau China; ^5^ Research Center of Clinical Medicine Affiliated Hospital of Nantong University Nantong Jiangsu China

**Keywords:** rat burn model, scar, S‐propargyl‐cysteine, wound healing

## Abstract

Hydrogen sulfide for wound healing has drawn a lot of attention recently. In this research, the S‐propargyl‐cysteine (SPRC), an endogenous H_2_S donor, was loaded on carbomer hydrogel, and a copper sheet rat burn model was developed. Pathological changes in rat skin tissue were examined using hematoxylin–eosin (HE) and Masson staining. The immunohistochemistry (IHC) staining was performed to detect the expression of Collagen I (Col I) and Collagen III (Col III). The mRNA levels of interleukin (IL)‐6, Col Iα2, Col IIIα1, tissue inhibitors of metalloproteinase (TIMP)‐1, matrix metalloproteinase (MMP)‐9, vascular endothelial growth factor (VEGF), and transforming growth factor (TGF)‐β1 were examined by quantitative real‐time chain polymerase reaction. The findings demonstrated that the collagen layer was thicker in the SPRC group during the proliferative phase, SPRC hydrogel promoted VEGF expression. In the late stage of wound healing, the expression of IL‐6, TIMP‐1, MMP‐9, and TGF‐β1 was inhibited, and the Col I content was closer to that of normal tissue. These results surface that SPRC hydrogel can promote wound healing and play a positive role in reducing scar formation. Our results imply that SPRC can facilitate wound healing and play a positive role in reducing scar formation.

## Graphical abstract

S‐propargyl‐cysteine (SPRC) smart transdermal delivery promotes wound healing in the animal model. SPRC smart transdermal delivery inhibits the proinflammatory factor IL‐6; downregulates the expression of TIMP‐1, TGF‐β1, and Collagen I; and upregulates the expression of VEGF. These effects suggest that it may serve as a treatment method for pathological scarring.

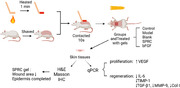

## 1 INTRODUCTION

The skin barrier aids in the body's defense against pathogenic microbes from the outside and helps to keep the inside environment stable.^1^ Common skin injuries that significantly disrupt people's lives include burns,^2^ diabetic ulcers,^1^ and traumatic wounds. Burn is a common injury to the skin or other organic tissue caused by various etiologies, such as thermal injuries, electrical injuries, chemical injuries, and so on.^2^ The World Health Organization estimates that 180,000 people die from burns each year, most of which occur in developing countries.^3^ Burns are common daily and impact people's physical and mental health by causing scarring, disability, and even life threatening. The systemic inflammatory response from severe burns can lead to immunological imbalances, respiratory distress syndrome, metabolic abnormalities, end‐organ hypoperfusion, and systemic hypotension.^2^

According to classical theory, the three consecutive stages of inflammation, proliferation, and regeneration comprise the necessary and challenging skin wound healing process.^4^, ^5^ After burn injury, various intracellular and intercellular pathways are activated immediately to repair the destruction of skin tissues, including the immune system, the blood coagulation cascade, and the inflammatory pathways.^6^, ^7^ In the second stage, proliferation, fibroblasts are functionally expressed, and granulation tissue is formed to replace the damaged tissue. During the regeneration phase, the body's natural mechanisms come into play to restore the tissue to its normal functional state. Blood vessels are regenerated, and the excess extracellular matrix (ECM) is degraded. While wound healing is frequently abnormal including immune disorders, upregulated fibroblast function, and excessive ECM deposition. Collagen‐rich ECM deposition typically signifies skin fibrosis, which results in pathologic scarring.

The treatment of skin injuries and scarring has always attracted a lot of attention.^8^, ^9^ One instance is the application of the basic fibroblast growth factor (bFGF),^10^ which can promote the expression of various growth factors. In treating clinical wounds, bFGF formulations, such as gels and sprays, have been utilized extensively. Despite the fact that novel high‐end dressings such as BFGF hydrogel have been widely used in the clinic, there is no satisfactory treatment for the scarring caused by skin damage.^11^ Therefore, it is of great significance to explore efficient options for promoting wound healing and improving scarring. In recent years, hydrogen sulfide has received increasing attention as a star molecule for its role in wound healing.

H_2_S is an endogenous gas transmitter that regulates the cardiovascular, neurological, gastrointestinal, and renal systems, significantly impacting inflammatory and immunological responses.^12^, ^13^ Additionally, H_2_S donors have been utilized to improve wound healing^14^ and reduce scarring.^15^ The enzymes cystathionine beta‐synthase (CBS), cystathionine gamma‐lyase (CSE), and 3‐mercapto pyruvate sulfurtransferase are involved in the synthesis of H_2_S.^16^ Among the many hydrogen sulfide donors, the S‐propargyl‐cysteine (SPRC; as named ZYZ‐802) is an endogenous H_2_S donor which increases the H_2_S levels in vivo through CSE/H_2_S pathway. In our group, the pharmacological effects of SPRC have been reported. It has excellent cardio‐protection and proangiogenesis, anti‐inflammation, and neuroprotection, showing potential clinical value in myocardial infarction, atherosclerosis, hypertension, rheumatoid arthritis, and neurodegenerative disease.^13^, ^17^, ^18^ A clinical trial application for SPRC‐Capsules as a new Class I medicine for the treatment of rheumatoid arthritis was submitted to the National Medical Products Administration in 2023. The researcher of this article hypothesized that the SPRC might improve wound healing and play an anti‐inflammatory role in burns, thus inhibiting the formation of scars.

This article tests this hypothesis. Commercial bFGF gel was employed as a positive control in this study to help clarify the role of SPRC. This paper represents the first attempt to formulate it as a carbomer hydrogel formulation and use it in skin injury restoration. In addition, a preliminary analysis of its mechanism of action has also been conducted. SPRC hydrogel offers a new strategy for skin wound healing and prevention of scarring.

## 2 RESULTS

### 2.1 Skin wound healing rates were over 96% in the SPRC group

**FIGURE 1:**
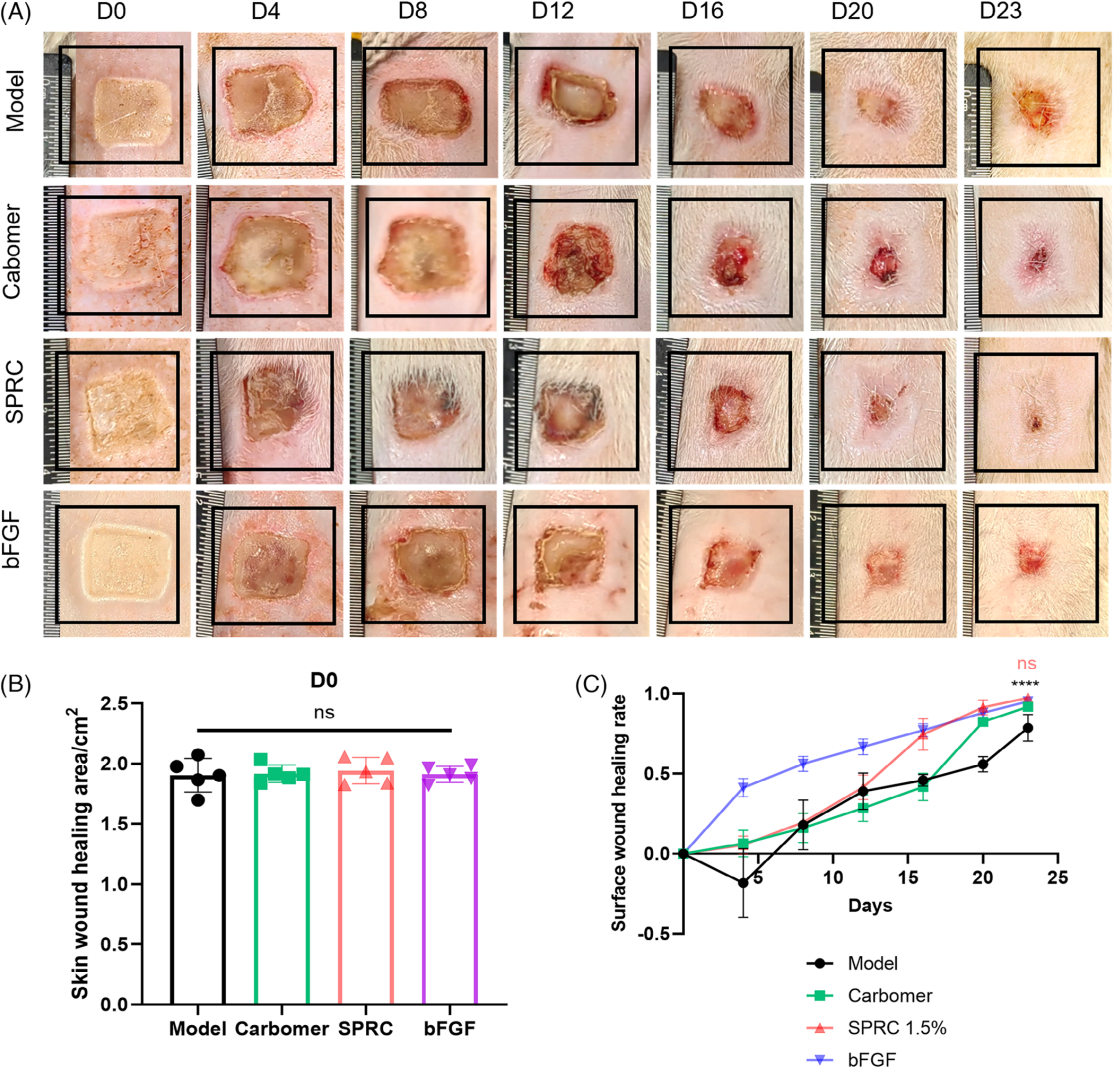
The therapeutic effect of 1.5% SPRC hydrogel in a rat burn model (length of a square = 20 mm, *n* = 5). (A) The photographs of each wound treated with model (without any treatment), carbomer (carbomer hydrogel), SPRC (1.5%(w/w) SPRC‐carbomer hydrogel), and bFGF. And each group from left to right is days 0, 4, 8, 12, 16, 20, and 23. (B) Data on the wound area for every group on the modeling day. There was no statistical difference in wound area between the groups. (C) The line chart of rats’ skin wound healing rate in each group. Between the SPRC and bFGF groups, there was no statistically significant difference in the rates of skin wound healing. However, the previous model groups and the SPRC differed significantly and *p* < 0.0001 is represented by “****.”

In order to verify whether SPRC hydrogel had a promoting effect on wound healing, a rat scald model was established. SPRC was loaded by carbomer‐based hydrogel to burn sites on Sprague–Dawley (SD) rats and comparing them with normal rats. The carbomer hydrogel group was constructed and treated with free‐SPRC carbomer hydrogel to exclude carbomer‐based effects. Photos taken of each rat during the experiment were shown every 4 days (days 0, 4, 8, 12, 16, 20, and 23; Figure 1A assesses the progress of skin wound healing in each group of rats. Figure 1B shows that on the day of modeling, there was no difference in the wound area across the groups, indicating that the modeling was successful.

The results of the wound healing rate are shown in Figure 1C. The region of skin damage has the propensity to grow after the molding has begun. After 3 days, the area around the wound displayed a noticeable inflammatory response; after 4 days, the size of the skin wound steadily reduced. The experiment's end was set at 23 days, depending on how quickly the wound healed. Treatment consists of putting hydrogel preparation on the area without debriding it, allowing the blood scab to fall off naturally. The experimenter discovered that the rats had scratching behavior on the wound that would result in varying degrees of blood scab shedding. In order to simplify the analysis of influencing factors, rat behavioral science was not discussed in this paper. As shown in Figure 1, the skin wound healing rate in the SPRC group was not significantly different from that in the bFGF group, despite both being superior to the model and carbomer group. There is preliminary evidence that SPRC hydrogel can speed up wound healing. Overall, these results provide preliminary confirmation that SPRC promotes wound healing.

### 2.2 SPRC hydrogel promoted skin tissue recovery in rats

**FIGURE 2:**
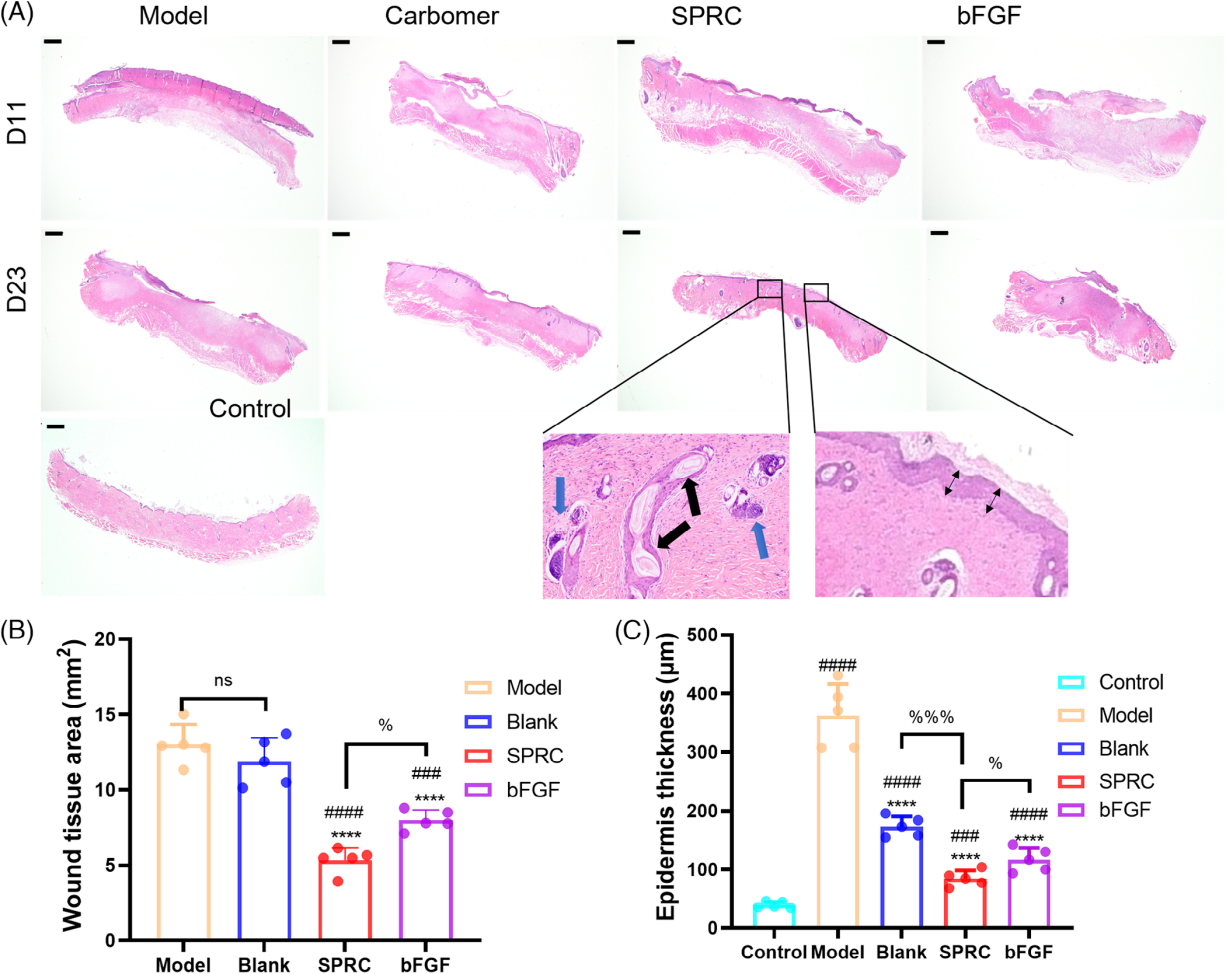
Representative histological appearance in each treatment group at D11 and D23 (*n* = 5). (A) Light micrographs of the HE‐stained slices (10×), scar bar = 1 mm. The blue arrows sebaceous glands, the black arrows indicate hair follicles, and the bi‐directional arrows represent epidermis thickness; (B) statistical plot of the wound tissue area of the sections (“*” indicates a statistically significant difference from the model group, “#” indicates a comparison with the carbomer group, and “%” indicates a comparison with the SPRC group.); (C) statistical plots of the new epidermal thickness (“*” indicates a statistically significant difference from the model group, “#” indicates a comparison with the control group, and “%” indicates a comparison with the SPRC group.).

**FIGURE 3:**
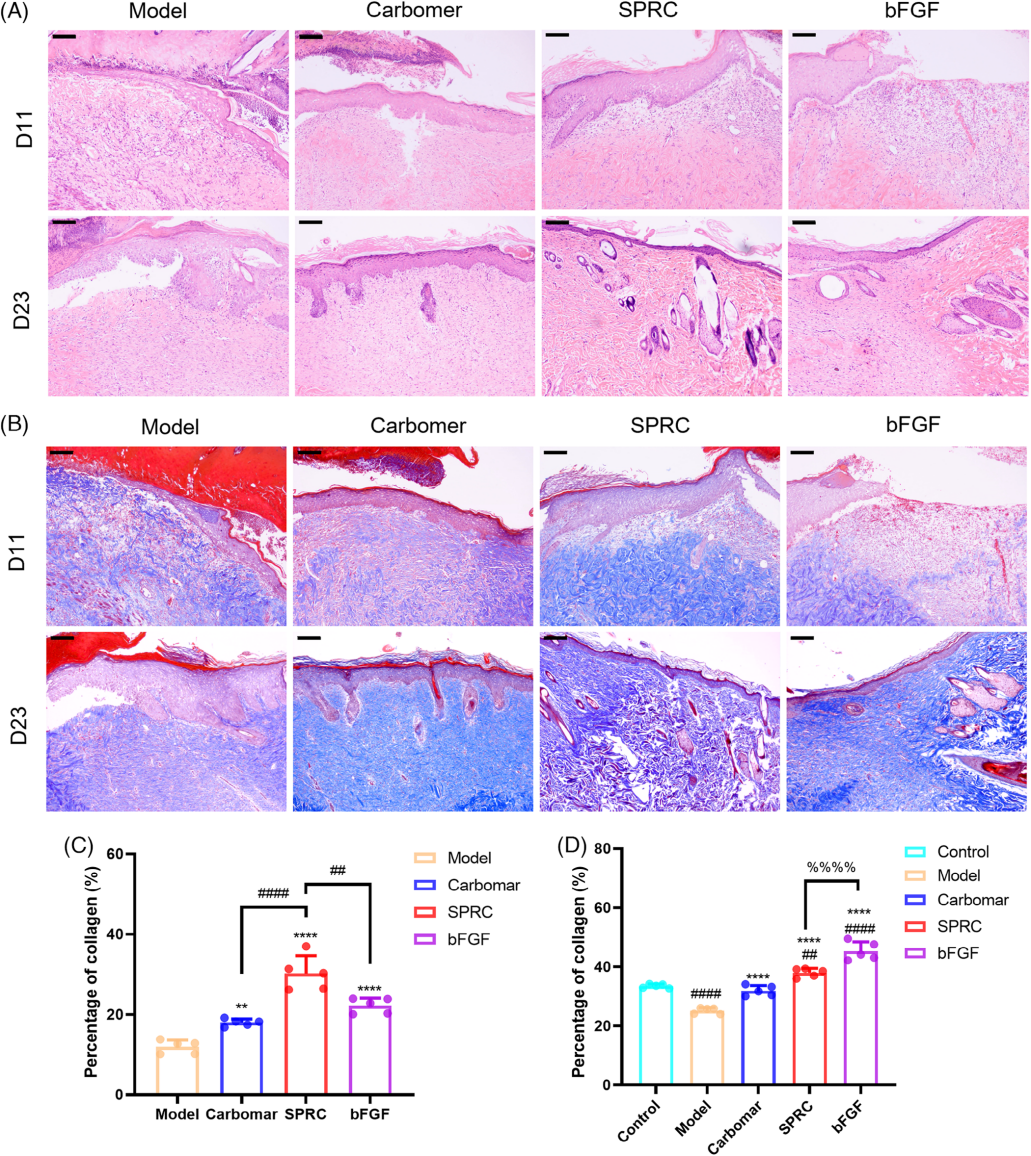
Results of H&E and Masson staining sections in SPRC treatment at D11 and D23 (*n* = 5). (A) Light microscope photograph of HE‐stained sections of D11 and D23 (100×, scar bar = 200 μm); (B) Light microscope photograph of Masson‐stained sections of D11 and D23 (100×, scar bar = 200 μm). Both the SPRC and bFGF groups had already begun to manufacture new epidermis; the SPRC group had a higher collagen content; the SPRC and bFGF groups had developed mature skin appendages, and the model group had the highest inflammation; the SPRC group had fewer collagen bundles parallel to the epidermis, which was closer to normal skin. (C) Collagen fibers area count graph for day 11 (“*” indicates a statistically significant difference from the model group, “#” indicates a comparison with the SPRC group.); (D) collagen fibers area count graph for day 23. (“*” indicates a statistically significant difference from the model group, “#” indicates a comparison with the control group, and “%” indicates a comparison with the SPRC group.)

After applying SPRC hydrogel, pathological alterations in skin burns were observed using histological analysis. Burnt skin tissues were collected on days D11 and D23 and stained with hematoxylin–eosin (HE) (Figure 2) and Masson (Figure 3) to examine histological alterations on the wound area. Pathological sections demonstrate the real process of healing a wound.

On day 11, all groups had inflammation, while the SPRC group had reduced inflammation; gradually, a thicker epidermis was grown, and the blood crust on the skin had not yet been eliminated, as can be seen in the 100× images of the D11 HE‐stained sections (Figure 3A). According to Masson staining (Figures 3B and C), the SPRC group's collagen content was much higher than that of the bFGF group model group and the carbomer group.

On day 23, the epidermis of the model and carbomer groups was not entirely covered, whereas the epidermis of the SPRC and bFGF groups was largely covered, and the wound closed (Figure 2A). Measurements of the epidermal thickness and wound tissue area were made. The SPRC group had more hair follicles and sebaceous gland growth than the bFGF group (as shown in Figures 2A and 3C). Figure 2B shows wound tissue area in each group: the SPRC < bFGF < carbomer < model group, and epidermal thickness: the SPRC < bFGF < carbomer < model group (as shown in Figure 2C). In addition, the SPRC group's collagen fibers content was higher than that of the control group and lower than that of the bFGF group (Figure 3D). These suggest that SPRC promotes wound healing more successfully and that the skin in the SPRC group recovered more closely to normal skin.

### 2.3 Collagen I expression was downregulated in the SPRC group

**FIGURE 4:**
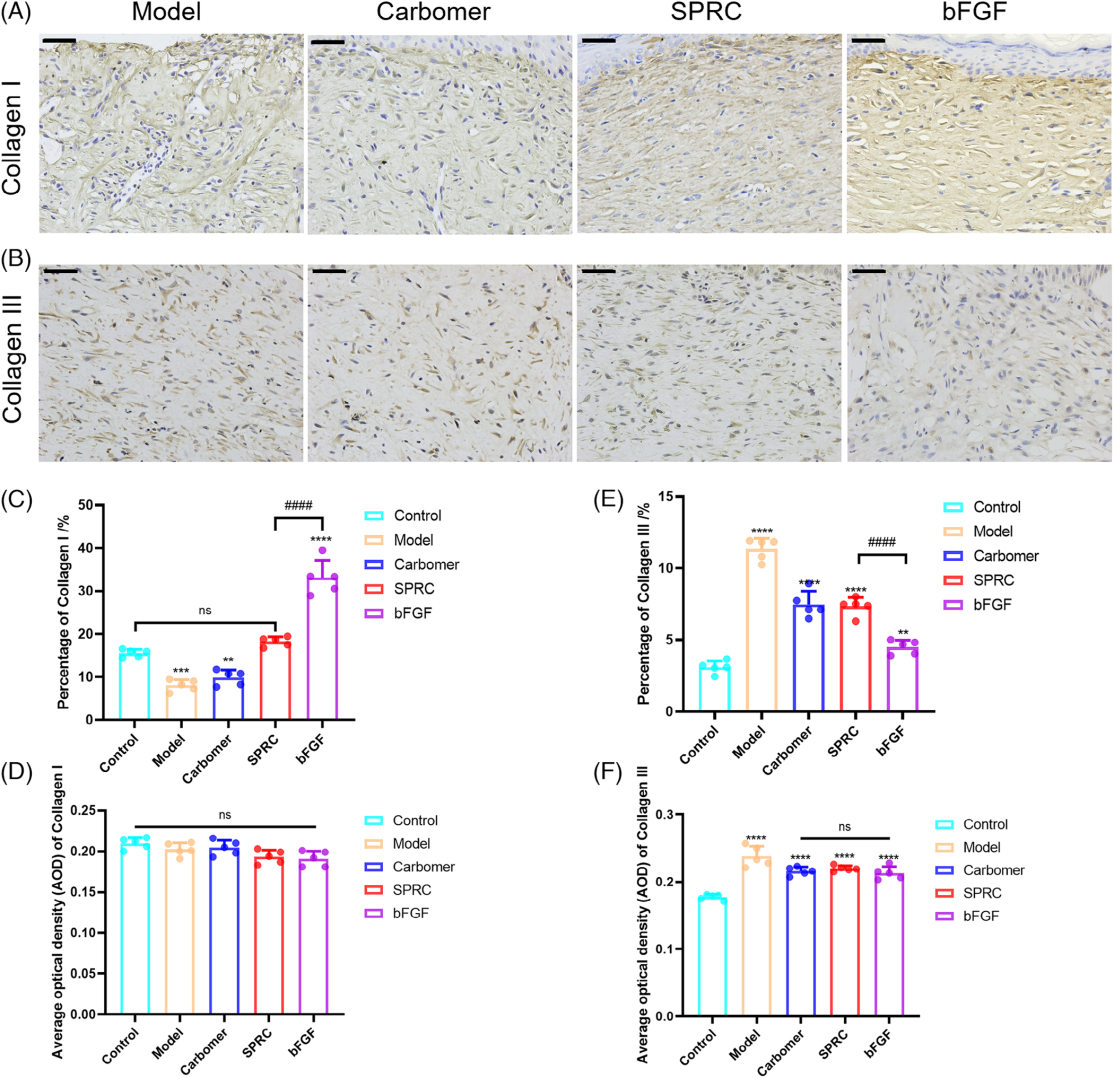
Results from immunohistochemistry showed that rats’ skin tissue on day 23 after burning expressed collagen types I and III (*n* = 5). (A) Micrographs of immunohistochemically stained sections of collagen I (400×, scar bar = 50 μm); (B) micrographs of the immunohistochemistry collagen III stained sections (400×, scar bar = 25 m); (C) statistics of the percentage of collagen I‐positive tissue in each group of rats’ skin; (D) statistics of the average OD of collagen I; (E) the percent‐age of Collagen III positive region in the rat skin tissues in each group; and (F) the AOD value of the immunohistochemical Collagen III stained sections. (*p* < 0.05 for statistical difference, “*” indicates a statistically significant difference from the control group, and “#” indicates a comparison with the SPRC group, *n* = 5.)

The primary components of the ECM include collagen and other substances. To determine the influence of SPRC hydrogels on Collagen I (Col I) and Collagen III (Col III) expression at the late stage of healing, IHC staining were utilized. In the animal studies described in this paper, the wounds in the SPRC and bFGF groups had closed after 23 days. There was no statistically significant difference between the SPRC group's Col I content and that of the normal control group, whereas the bFGF group's Col I expression (Figures 4A and C) was higher than that of normal skin. And there was no statistically significant difference in the average optical density (AOD) values of Col I in each group (Figure 4D). The content of Col III (Figures 4B and E) was higher in all experimental groups than in the control group, and the expression was highest in the model group and lowest in the bFGF group. The AOD value of Col III (Figure 4F) was lowest in the control group and highest in the model group, with no statistical difference in the carbomer, SPRC, and bFGF groups. According to these findings, the SPRC and bFGF groups had entered into the remodeling phase, whereas the model and carbomer groups are still in the proliferative stage. Col I content was lower in the SPRC group, which helped to prevent scarring.

### 2.4 SPRC promoted the mRNA level expression of vascular endothelial growth factor

**FIGURE 5:**
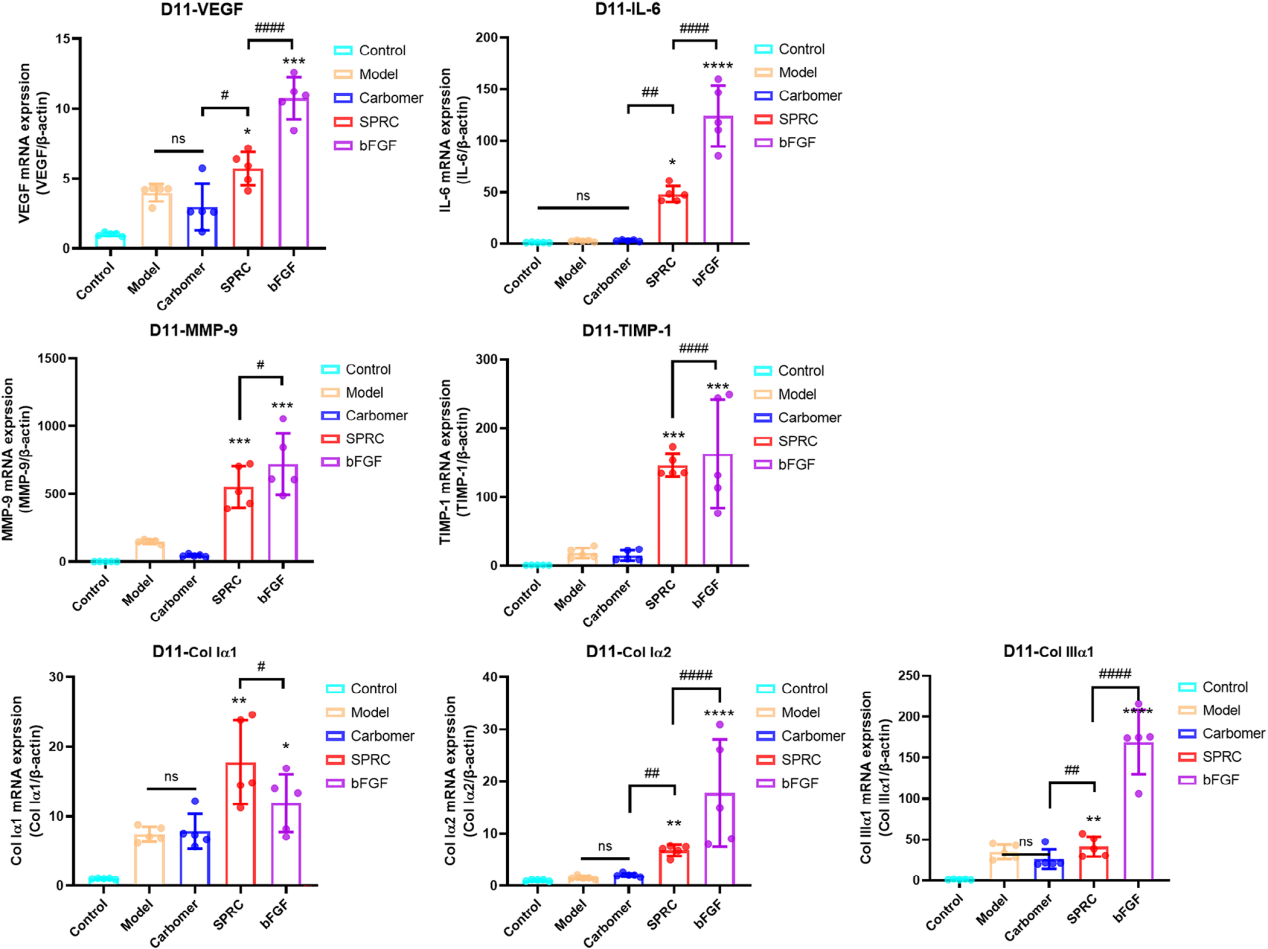
The outcomes of each group's VEGF, IL‐6, TIMP‐1, MMP‐9, Col Iα1, Col Iα2, and Col IIIα1 mRNA expressions on day 11. “*” denotes a comparison with the model group, and “#” denotes the SPRC group, *n* = 5.

mRNA was collected from the wound tissue of each group (days 11 and 23) to look for the expression of vascular endothelial growth factor (VEGF), interleukin (IL)‐6, Col Iα2, Col IIIα1, tissue inhibitors of metalloproteinase (TIMP)‐1, and matrix metalloproteinase (MMP)‐9. This was done to further examine the role of SPRC in promoting wound healing. According to the quantitative polymerase chain reaction (qPCR) results, both the SPRC and bFGF groups had higher levels of IL‐6, Col Iα1, Col Iα2, Col IIIα1, TIMP‐1, MMP‐9, and VEGF expressions on day 11 (Figure 5), which aided fibroblast migration and proliferation and accelerated wound healing.^19^ These findings show that SPRC hydrogel enhanced wound healing by increasing VEGF expression.

### 2.5 SPRC inhibited the mRNA level expression of IL‐6, transforming growth factor‐β1, and TIMP‐1

**FIGURE 6:**
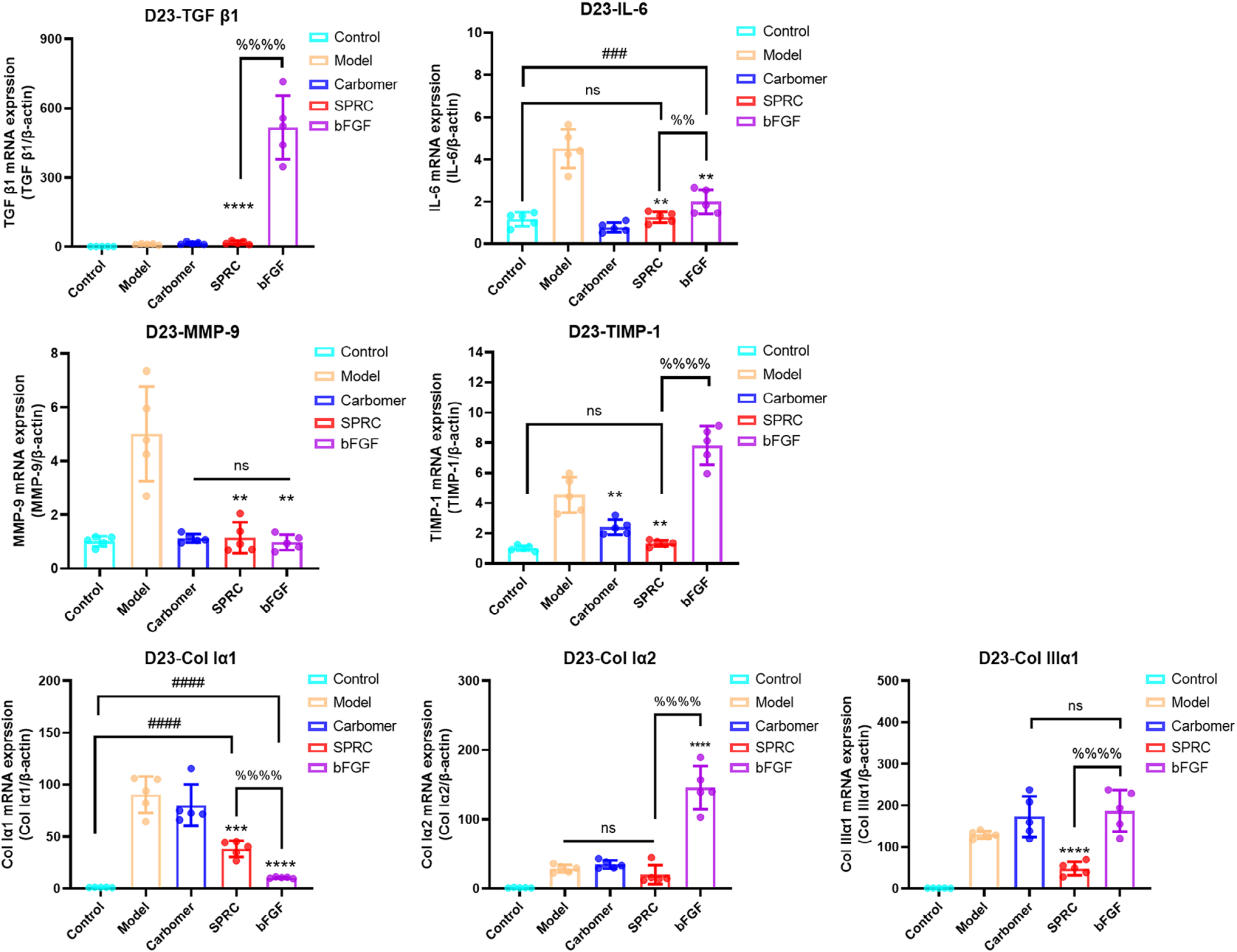
The outcomes of each group's TGF‐β1, IL‐6, TIMP‐1, MMP‐9, Col Iα1, Col Iα2, and Col IIIα1 mRNA expressions on day 23. “*” denotes a comparison with the model group, “#” denotes the control group, and “%” denotes the SPRC group, *n* = 5.

It is generally accepted that increased transforming growth factor (TGF)‐β1 expression is a hallmark of fibrosis,^20^ and inflammation also leads to scar formation.^21^ Therefore, in order to investigate whether SPRC could reduce scarring, this paper investigated the mRNA expression of IL‐6, MMP‐9, TIMP‐1, Col Iα1, Col Iα2, Col IIIα1, and TGF‐β1 at the regeneration period. At day 23 (in the regeneration phase, shown in Figure 6), IL‐6 expression remained upregulated in the bFGF group relative to the control group, but it considerably decreased in the SPRC group. While TIMP‐1 expression remained upregulated in the bFGF group, which is not conducive to excessive ECM degradation and is prone to a scar, MMP‐9 and TIMP‐1 expression in the SPRC group were not statistically significant when compared with the control group. TGF‐β1 expression, one of the crucial indicators of fibrosis,^22^ was also not statistically different between the SPRC and control groups, but it was increased in the bFGF group, and the Masson staining (Figure 3D) confirmed these findings. In comparison with the SPRC and control groups, the expressions of Col Iα2 and Col IIIα1 were also higher in the bFGF group. These studies surfaced that SPRC can follow the inhibition of TGF‐β1 and TIMP‐1 expression during remodeling, which favored the degradation of excessive ECM and contributed to the reduction of scarring.

## 3 DISCUSSION

The authors of this research believe that the indicators for effective wound healing must be comparable to those of normal rats. Figure 1A depicts how the wounds changed over time in each group of rats. The skin wound healing rate of the SPRC group was higher than that of the model and carbomer groups, was not statistically different from that of the bFGF group, and appeared capable of promoting wound healing. In the early inflammatory phase of a wound, neutrophil activation and migration may cause wound expansion.^23^ As a result, on day 4 of our research, the skin wound healing rate is negative. During the first 15 days of the experiment, the skin wound healing rate was much higher in the carbomer group than in the SPRC group, most likely as a result of the time it took for the blood scab to break off. In this study, there was no debridement procedure performed to control factors. Rat behavior was not covered in this study to streamline the analysis of the factors that affected the experiment. By using histology, immunohistochemistry, and qPCR, we also looked at the molecular mechanisms and wound pathology in the therapy of SPRC.

SPRC is an endogenous H_2_S donor which increases the H_2_S levels in vivo through CSE/H2S pathway. SPRC has been shown to promote angiogenesis in models of myocardial ischemia, activating VEGF expression by acting on STAT3 and providing a new target for ischemia/reperfusion heart disease therapy.^24^ Our group has also developed a novel liposome to load SPRC, which inhibits myocardial fibrosis by inhibiting the TGF‐β1/Smad signaling pathway.^25^ As an anti‐inflammatory compound, SPRC attenuates inflammatory symptoms, such as the downregulation of IL‐6 and tumor necrosis factor‐α in adjuvant‐induced arthritis rats by regulating gut microbiota.^26^ In an in vivo model of acute pancreatitis in mice, SPRC significantly inhibits proinflammatory cytokines (IL‐1β and IL‐6) and augments anti‐inflammatory cytokine (IL‐10).^27^ A recent study has reported that SPRC restrains the progression of rat periodontitis by regulating the Th17/Treg balance by inhibiting the ERK/CREB pathway.^28^ This work described the first‐ever application of SPRC—loaded with carbomer hydrogel—for the purpose of repairing skin injuries. Carbomer hydrogel has gained popularity in clinics in recent years and can deliver drugs in a controlled manner over time.

According to histological findings, in the SPRC and bFGF groups, the epidermal growth had finished by day 23 following damage, and the formation of hair follicles, sebaceous glands, and other cutaneous appendages had started. However, the model group and carbomer group displayed incomplete epidermal development, and H&E‐stained sections revealed that there was still an inflammatory response, and their wound healing process was slower than that of the drug groups.

D11 and D23 in this study correspond to the proliferation and regeneration phases of wound healing, respectively. After a skin injury, neutrophils react quickly, go to the area, and release a lot of inflammatory signaling molecules, such as IL‐6, and promote the growth of fibroblasts, which produce ECM, including collagen,^29^ which is consistent with the outcomes of our experiment. Blood vessels and lymphatic vessels can be produced more quickly because of the growth factor VEGF.^30^ The expression of VEGF reflected wound healing and promoted angiogenesis.^31^ In our results, IL‐6, MMP‐9, TIMP‐1, Col Iα2, Col IIIα1, and VEGF levels were increased in the SPRC and bFGF groups at day 11, which aided in fibroblast migration and proliferation.^32^ These results suggest that SPRC can indeed promote wound healing. Col III is degraded throughout the remodeling process, while collagen type I is gradually formed. This process is crucial for maintaining healthy tissue and allowing for its proper functioning.^21^

Inside the environment, homeostasis is supported by ECM, which speeds up wound healing and promotes tissue cell proliferation.^29^ Masson staining revealed that collagen expression was higher in the SPRC group than in the SPRC group on day 11, but qPCR results revealed that Col I and Col III mRNA expression was lower than in the bFGF group. The authors suggest that the peak expression in the SPRC group was reached before day 11, which indirectly suggests that SPRC promotes the wound‐healing process. Figure 3A, H&E staining, demonstrated that the SPRC and bFGF groups' wound tissue had grown a thicker epidermis and that epidermal proliferation suggested wound healing.^33^ Col III acts mainly in the early stages of wound healing, expressing more than Col I and influencing its formation,^34^ consistent with the results in Figure 5. In the later stages of wound healing, mature Col I gradually replace Col III.^21^ An increased amount of Col I was found in pathological scar tissue.^35^ In contrast to the bFGF group on the 23rd day, the SPRC group exhibited lower levels of Col type I, according to the IHC results. It can be concluded that SPRC works better at improving scarring than bFGF does. Col III content in each group was higher at day 23 than in the control group, which is understandable that the wounds in each experimental group had not yet entirely healed.

At 23 days, there was no difference in the expression of MMP‐9, TIMP‐1, and TGF‐β1 between the model, carbomer, and control groups, and the rat skin itself was not prone to keloid development. A study has shown that inhibiting the expression of IL‐6 can effectively downregulate systemic inflammation and control burn injury.^4^ According to our findings, IL‐6 mRNA expression was lower in the SPRC group than in the model or bFGF group. This is in line with earlier reports that SPRC can lower IL‐6 levels.^26^ Collagen was still expressed in the model and carbomer groups because the wounds had not yet healed. qPCR results showed that TGF‐β1 mRNA expression in the SPRC group was not statistically different from that in the control group, whereas in the bFGF group, TGF‐β1 was overexpressed. In this study, the downstream signal molecules MMP‐9 and Col I were downregulated and not statistically different from the control group. Col I expression was lower than in the bFGF group. Findings in this article are similar to earlier research^17^ in our group that showed SPRC can inhibit the TGF‐β1/Smad signaling pathway, hence reducing the expression of MMP‐9 and Col I. According to Hu et al.,^22^ inhibiting the TGF‐β1/Smad signaling pathway effectively prevented tissue fibrosis. Furthermore, keloid fibroblasts produce excess collagen, and TGF‐β1 induces the expression of Col III.^36^ At the same time, the mRNA expression levels of Col Iα2 and Col IIIα1 in the SPRC group were fairly lower than that in the bFGF group. Therefore, our findings imply that SPRC may have an improvement in pathological scarring.

Rapid ECM production during the proliferative phase benefits wound healing, whereas excessive dysregulation of ECM breakdown during the recovery phase results in the development of keloid lesions.^21^ Figure 6 from this study's findings shows that MMP‐9 and TIMP‐1 mRNA expressions in the SPRC group were similar to that of the control group, and TIMP‐1 expression was lower than the bFGF group. Wang et al.^37^ reported that the downregulation of TIMP‐1 contributes to the reduction of scar formation. In addition, the balance of MMP9 and TIMP‐1 expression is important for skin homeostasis.^38^ This further demonstrates that SPRC can improve scarring.

The findings of this study demonstrate that SPRC hydrogel both facilitates wound healing and improves scarring, which has never been reported before. This study enriches the clinical utility of the SPRC. While the role of SPRC in wound healing and improving scarring still needs to be validated and explored in more trauma models in the future.

## 4 CONCLUSIONS

This study broadens the delivery route and application of SPRC. SPRC‐hydrogel can promote the healing of wounds. In the proliferation phase, it can boost the expression of VEGF and collagen and create a favorable environment for cell growth. During remodeling, it may effectively reduce IL‐6, TGF‐β1, MMP‐9, Col I, and TIMP‐1, preventing ECM overgrowth. SPRC hydrogel offers a new and effective strategy for treating skin damage and actively improves scarring. In the future, these findings still need to be validated in more skin damage models.

## 5 MATERIALS AND METHODS

### 5.1 Chemical materials

The chemical materials used in this study include SPRC (also named ZYZ‐802, Shanghai), carbomer 940 (Macklin, China), sodium carboxymethyl cellulose (Macklin), glycerin (Macklin), sodium hydroxide (Macklin), Tween‐20 (Sigma, USA), Tween‐80 (Macklin), TRIzol reagent (Carlsbad, CA, USA), and phosphate‐buffered saline (PBS) tablets (Gaithersburg, MD, USA). Recombinant bovine bFGF was purchased from Essex Bio‐Technology (Zhuhai, China). Hydrogen peroxide solution was obtained from ANNJET (Shandong, China). Alcohol, n‐butanol, and xylene were purchased from SINOPHARM (Shanghai, China). Masson Tricolor Staining Kit, HE Staining Kit, BioDewax and clear solution, tris‐ethylene diamine tetraacetic acid antigen retrieval solution (Tris‐EDTA), pH = 9.0), bovine serum albumin (BSA), horseradish peroxidase (HRP) conjugated goat anti‐rabbit IgG (H+L), and DAB chromogenic kit were purchased from Servicebio (Wuhan, China). Total RNA was extracted with RNA Extraction Kit was purchased from Solarbio (Beijing, China). The HiFiScript gDNA Removal cDNA Synthesis Kit was obtained from CWBIO (Jiangsu, China). The PerfectStar Green qPCR SuperMix (+Dye I/+Dye II) was purchased from TRANS (Beijing, China).

### 5.2 Animals

Adult male SD rats (250−300 g, 4−6 weeks) were purchased at the Chinese University of Hong Kong and raised at the Animal Laboratory of the School of Pharmacy, Macau University of Science and Technology. The rats were kept at the room temperature and humidity‐controlled animal facility with a 12‐h light/dark cycle. All animal studies described herein were approved by the Animal Care and Use Committee of the Municipal Affairs Bureau of Macau (approval number AL010/DICV/SIS/2018).

### 5.3 Rat burn model

The rat burn model was built with a brass sheet. Rats were deeply anesthetized with freshly prepared sodium pentobarbital solution (2%, 3 mL/kg) by intraperitoneal injection to relieve suffering. The back hair of the rats was removed. An 8 × 10 mm brass piece was heated with an alcohol lamp external flame for 1 min and placed on the exposed skin of the rats for 10 s under the same pressure. (Before building the model, the experimenter has practiced repeatedly to get the same pressure.)

For the mechanism study, SD rats were randomly divided into five groups (group labelling, number):

(1) control group (control, normal rats, *n* = 5),
(2) model group (model, *n* = 10, one died because of intraperitoneal injection),
(3) model + carbomer group (carbomer, *n* = 10, one died because of intraperitoneal injection),
(4) model + SPRC group (SPRC, *n* = 10),
(5) model + bFGF positive control group (bFGF, *n* = 10).

Five rats from each group were selected and euthanized on 11 or 23 postinjury days, labeled as D11 and D23, respectively. The wound and a small amount of peripheral normal skin tissues were obtained from the euthanized rats. Each rat skin tissue was washed with sterilized PBS solution. Half of each skin tissue was fixed in 4% paraformaldehyde for histopathological analysis and another skin tissue was frozen in liquid nitrogen and stored in a −80°C refrigerator for molecular mechanism studies.

### 5.4 Preparation of hydrogel preparations

#### 5.4.1 Preparation of carbomer hydrogel base

The carbomer 940 powder (400 mg) was weighed and put into a glass beaker with a magnetic stirrer, then treated with distilled water (20 mL) and stirred continuously standing to the full swelling at 50°C water bath. Sodium hydroxide (160 mg, 4 mmol) was dissolved in distilled water (4 mL). The sodium hydroxide solvation was added drop by drop to the swollen carbomer solution (pH = 5). The carbomer solution was gradually transparent and viscous and formed, and the final pH = 7 to obtain the hydrogel base, which was colorless and transparent.

#### 5.4.2 Preparation of carbomer hydrogel

The Tween‐20 (200 mg), Tween‐80 (200 mg), and glycerin (2 g) were added into the hydrogel base (which was obtained in Section 4.4.1) with stirring. The 0.5% sodium carboxymethyl cellulose solution (5 mL) was poured into the above hydrogel. Distilled water was added to the hydrogel until the expected total mass was 40 grams and stirred continuously to mix thoroughly. The colorless and transparent carbomer hydrogel agent (pH = 7) was gained and stored in 4°C.

#### 5.4.3 Preparation of SPRC hydrogel

The SPRC powder (600 mg) was weighed and solved in distilled water with a magnetic stirrer at room temperature. Tween‐20 (200 mg) and Tween‐80 (200 mg) were added to the SPRC solution while stirring constantly. All of the above system and glycerin (2 g) was added to the hydrogel base (has been configured in 4.4.1) in batches with stirring constantly for 5 min to mix thoroughly. Distilled water was added to the hydrogel until the expected total mass was 40 grams and stirred continuously to mix thoroughly. Finally, the 1.5% (w/w) SPRC hydrogel preparation (pH = 7) was obtained and stored in 4°C.

### 5.5 The treatment of rat burns

The SD rats of the control group were fed usually without any injury. The model group was burned without any treatment. In the carbomer group, rats were scalded and treated with carbomer hydrogel (prepared in Section 4.4.2.), and the SPRC group was treated with 1.5% (w/w) SPRC hydrogel. The bFGF group, as positive drug control, was treated with commercial Recombinant bovine bFGF hydrogel. Each hydrogel was applied 100−200 μL equably to the wound and normal tissue around it. Each hydrogel was administered daily from 18:00 to 21:00 once a day during the uninterrupted experimental period.

### 5.6 Histopathological examination

The rat skin tissues (Section 4.3 mentions this) were paraffin‐embedded after being dried using a gradient concentration of alcohol. Using the appropriate Masson's trichrome or HE staining standard techniques, tissue slices measuring 3−4 microns were created. The tissue sections were photographed under a light microscope (Olympus, Japan).

The epidermal thickness was determined at five different sites in one section and averaged using ImageJ software after the D23 10× He‐stained images were enlarged. The area of granulation tissue was measured in 10X He‐stained sections of the D23. Prism software was used to analyze and plot the data.

### 5.7 Immunohistochemical analysis

Skin tissue sections (D23) were deparaffinized using a BioDewax and Clear Solution (10 min × 3) and anhydrous ethanol (5 min × 3). The skin tissue samples were subjected to heat‐mediated antigen repair in a Tris‐EDTA (1×, pH = 9.0). To inhibit endogenous peroxidase, the sections were submerged in a 3% hydrogen peroxide solution for 25 min at room temperature and protected from light, and then washed with PBS (pH 7.4). Dropwise additions of 3% BSA were used to cover the tissue uniformly in a histochemical circle, which was then sealed and left at room temperature for 30 min. The blocking solution was shaken off gently, the primary antibody (anti‐Col I, Abcam; anti‐Col III, Affinity) was dropped, and incubate the slice flat in a moist box at 4°C for the duration of the next day.

On a decolorization shaker, the slides are washed three times for 5 min in PBS (pH 7.4). The sections are shaken dry before being covered with a drop of HRP conjugated Goat Anti‐Rabbit IgG. They are then let to sit at room temperature for 50 min. When the positive was brownish‐yellow, the color development period was stopped by washing the sections with tap water under the microscope. To stop the color development, tap water was rinsed over the parts. After the cell nucleus had been re‐stained with hematoxylin dye, the slices were then dehydrated in gradient alcohol, n‐butanol, and xylene and then sealed. The sections were examined under a white light microscope (Olympus IX2‐SL, Japan) to determine the outcome.

### 5.8 RNA extraction and quantitative real‐time PCR

Skin tissue (obtained in D11 and D23, mentioned in Section 4.3) of the rat was removed at −80°C and weighed at 50–100 mg by the analytical balance in an RNA enzymes free tube (2 mL) and treated with 1 mL TRIzol Reagent. Total RNA was extracted with RNA Extraction Kit (Solarbio, Beijing) according to the manufacturer's protocol, and quantified by a NanoDrop 2000 spectrophotometer (Thermo Fisher Scientific, USA).

Following the manufacturer's process, one microgram of RNA was reverse transcribed by the HiFiScript gDNA Removal cDNA Synthesis Kit (CWBIO). Several transcripts were measured using PerfectStar Green qPCR SuperMix (+Dye I/+Dye II) (TRANS, China) through ViiA™ 7 Real‐Time PCR System (Applied Biosystems). Specific primers are listed in Table 1.

**TABLE 1:** Primer sequences for rats.

Rat genes	Primer sequences
β‐Actin	F: ATTGTAACCAACTGGGACG
	R: TCTCCAGGGAGGAAGAGG
IL‐6	F: CCTACCCCAACTTCCAATGCT
	R: GGTCTTGGTCCTTAGCCACT
Col Iα2	F: GGTCCAAGAGGAGAACGTGG
	R: GGGACCTCGGCTTCCAATAG
Col IIIα1	F: AGAGGCTTTGATGGACGCAA
	R: GGTCCAACCTCACCCTTAGC
MMP‐9	F: GATCCCCAGAGCGTTACTCG
	R: GTTGTGGAAACTCACACGCC
TIMP‐1	F: AGCCCTGCTCAGCAAAAGG
	R: CTGTCCACAAGCAATGACTGTCA
VEGF	F: TATGTTTGACTGCTGTGGACTTGA
	R: CAGGGATGGGTTTGTCGTGT
TGF‐β1	F: TACGCCAAAGAAGTCACCCG
	R: GTGAGCACTGAAGCGAAAGC

### 5.9 Statistical analysis

The size of the wound, epidermal thickness, positive area for IHC and Masson staining, and AOD values were calculated by ImageJ software (LOCI, University of Wisconsin). Additionally, the skin wound healing rates were calculated using the formula (1).

Wound healing rate (*D_n_*) = [skin wound area (*D*_0_) − skin wound area (*D_n_*)] × 100% (1)

Every data was shown as “mean ± SD (standard deviation).” The application GraphPad Prism 8.0.2 was used to perform the calculations (GraphPad Software Inc., San Diego, CA, USA). The differences between the groups were compared using a one‐way analysis of variance and Tukey's post hoc multiple comparisons. *p* < 0.05 values exhibited statistical significance.

## AUTHOR CONTRIBUTIONS

*Conceptualization*: Y. C. and Y. Z.; *methodology*: X. Z., Y. C., and Z. L.; *validation*: X. Z., Y. C., X. J., B. S., and L. W.; *formal analysis*: X. Z., Y. C., and Z. L.; *investigation*: X. L. and M. Y.; *resources*: K. C. and M. Z.; *data curation*: X. Z. and X. J.; *writing—original draft preparation*: X. Z.; *visualization*: X. J. and X. L.; *supervision*: Y. C. and Y. Z.; *funding acquisition*: Y. Z. All authors have read and agreed to the published version of the manuscript.

## CONFLICT OF INTEREST STATEMENT

The authors declare no conflict of interest.

## ETHICS STATEMENT

This research was approved by the local ethics committee (Municipal Affairs Bureau of Macau) and all experiments were carried out according to the method approved (approval number AL010/DICV/SIS/2018).

## Data Availability

Raw data are available from the corresponding authors upon request.
